# Pathological Society

**Published:** 1896-11-07

**Authors:** 


### Medical Progress and Hospital Clinics.

[*The Editor will be glad to receive offers of co-operation and contributions from members of the profession. All letters should be addressed to* The Editor, at the Office, 28 & 29, Southampton Street, Strand, London, W.C.]

### PATHOLOGICAL SOCIETY.

At the meeting on November 3rd, Mr. Lockwood showed specimens of hydro-salpinx in which the tube at the uterine end was almost normal, but after a sudden bent was largely dilated in the shape of a retort. He argued against the suggestion that the adhesion of the ovary to the tube was due to an inflammatory process, maintaining that it was due to a spreading out of the layers of the broad ligament.

Mr. Lockwood also showed specimens of undescended testes, pointing out that in the performance of operations for the radical cure of hernia it was frequently necessary, or at least seemed desirable, to remove testes which lay in or about the ring, and that such retained testes were often so altered in character as to be incapable of performing their function of manufacturing spermatazoa. The President, Mr. Butlin, thought Mr. Lockwood would have to get over a difficulty arising from the fact of men with undescended testes being fathers of families. Mr. W. G. Spencer mentioned cases of retained testes, in which the epididymis was reduced to a few tubes only, and in which the testes were atrophied and incapable of function. Mr. Barker, however, thought we should not too readily assume that even a very small organ was functionally incapable, and he described a case in which a well-developed man had a mere rudimentary testis just outside each ring, yet he was the father of a family, and his virile power appeared to be retained in full vigour. He doubted whether these undescended testes were entirely devoid of function, and their removal should not be lightly undertaken.

Mr. J. Hutchinson, jun., described a case of rodent ulcer of the forearm, pointing out the rarity of the disease anywhere but on the face, and entering into the question of the point of origin of the disease, which he thought lay in the rete mucosum. This led to a very animated discussion. Dr. Kanthack maintained that if one wanted to know the origin of the disease one must examine cases before ulceration had taken place, and in every case which he had thus examined the growth began in the corium, and the rete mucosum had nothing to do with it. The hair follicles and sweat glands were unaffected, but the sebaceous glands had disappeared. Mr. Bowlby largely agreed with Dr. Kanthack. In early cases it was absolutely clear that the growth was beneath the cutis and epidermis, and it only appeared to grow from the rete Malpighii when it had already reached it, and then it was too late to see its origin. If sections were made early enough it was beneath the rete, and very likely occurred in the sebaceous glands. Mr. W. G. Spencer questioned the advantage of keeping up the term rodent ulcer, and thought that a slow growing epithelioma might have much the character of a rodent ulcer, and alluded to specimens which he had shown in regard to this point before another society. The President thought rodent ulcer and epithelioma quite distinct, and had never seen any transition forms between the two conditions. Mr. Shattock thought rodent ulcer was a pathological entity.

Mr. J. Hutchinson, jun., also showed a Chondrosarcoma of the Submaxillary Gland, a condition, however, which he thought was more fitly expressed by the term Adeno-chondroma.

Dr. Rolleston and Dr. Tooth both described very interesting cases; and Dr. Kanthack and Mr. E. H. Shaw showed specimens illustrating the Use of Formalin in the Preparation of Museum Specimens.

